# Quality of life among parents of autistic children: questionnaire validation study and multivariate analysis of associated factors

**DOI:** 10.3389/fpsyt.2025.1554368

**Published:** 2025-07-09

**Authors:** Łukasz Konowałek, Maja Kotowska-Bąbol, Jan Łukasik, Piotr Remiszewski, Tomasz Wolańczyk

**Affiliations:** ^1^ Department of Child and Adolescent Psychiatry, Medical University of Warsaw, Warsaw, Poland; ^2^ Department of Paediatrics, Medical University of Warsaw, Warsaw, Poland; ^3^ Faculty of Medicine, Medical University of Warsaw, Warsaw, Poland

**Keywords:** autism, parents, wellbeing, quality of life, children

## Abstract

Parents of children with autism often experience unique challenges that can impact their emotional wellbeing. The aim of this study was to validate the Polish adaptation of the Quality of Life in Autism Survey (QoLA) and to examine the factors influencing quality of life (QoL) among parents of children with autism spectrum disorder (ASD). The survey was administered to 372 participants, predominantly mothers, and assessed QoL using the QoLA-A and QoLA-B scales alongside established tools such as the Beck Depression Inventory and WHOQOL-BREF. Results showed high reliability for the QoLA-A scale, supported by Cronbach's alpha (0.93) and omega coefficients, and confirmed an 8-factor model consistent with Schalock's domains of QoL. The scale showed strong correlations with psychological health, social relationships and environmental factors, validating its criterion and construct reliability. Conversely, QoLA-B showed limitations, capturing only five dimensions and explaining less than 50% of the variance. Important predictors of parental QoL included employment status, material well-being and depressive symptoms, with externalising behaviours in children having a significant negative influence. Intellectual and communication disabilities, although a significant challenge, were not strongly correlated with parental QoL. The findings highlight the need to address socio-economic and mental health support for caregivers, with an emphasis on tailored interventions that enhance parental wellbeing, rather than exclusively targeting child-specific therapies. This comprehensive validation highlights the utility of the QoLA-A and the nuanced factors that influence caregiver QoL.

## 1 Introduction

### 1.1 Autism and parental quality of life

Autism spectrum disorder (ASD) is a complex neurodevelopmental condition. It is characterized by challenges with social interaction, and communication, as well as the presence of restricted, repetitive patterns of behaviour and interests (1). The prevalence of ASD has increased over time and currently affects approximately 1 in 36 children worldwide by the age of 8 years, with boys being significantly more affected than girls (2). Autism affects not only the individual, but also places significant challenges on their families and caregivers. A review of the literature on the quality of life of parents of children diagnosed with ASD reveals a consistent trend of lower satisfaction compared to parents of typically developing children (3). According to a recent meta-analysis involving 27,942 individuals, siblings of those diagnosed with ASD are more likely to experience adverse outcomes compared to siblings of individuals without ASD or with other disabilities (4). These outcomes include a higher likelihood of internalizing behavioural problems, limitations in social functioning, and symptoms of anxiety and depression compared to siblings of children with Down syndrome or those without disabilities.

In addition to reporting a lower quality of life in general, parents of children with ASD face specific challenges and elevated levels of stress compared to parents of typically developing children or those with other disabilities (5). These challenges include difficulties in accessing appropriate services and resources, managing problematic behaviours, navigating social interactions and relationships, and coping with the daily demands of parenting a child with ASD. Some studies have suggested that the stress experienced by these parents can negatively impact their overall mental and physical health (6). It is therefore unsurprising that viewing the family as an integrated unit and addressing their needs alongside those of the child can significantly enhance the effectiveness of therapy and improve overall family functioning (7).

### 1.2 Quality of life of parents of autistic children in Poland

Recent studies conducted in Poland confirm that parents of children with autism spectrum disorder report significantly lower quality of life across multiple domains. For example, research comparing parents of children with ASD to parents of neurotypical children revealed substantial differences in general health, mental health, social functioning, and vitality, with the ASD group scoring consistently lower (8). Similar findings were observed in studies analysing family functioning and stress coping strategies, which demonstrated reduced psychosocial well-being among parents of children with ASD (9). Moreover, a cross-cultural study comparing parents from Poland, Belarus, and France revealed that Polish parents reported the lowest overall quality of life according to the WHOQOL-BREF scale (10). These findings highlight the importance of using culturally sensitive and linguistically adapted tools for assessing parental quality of life in families affected by autism.

### 1.3 Measurement of quality of life

As crucial as it is, the concept of quality of life is often difficult to measure in a specific and consistent manner. Some authors even argue that it is not possible to measure comprehensively (11). However, researchers looking for useful models to provide effective care for individuals with ASD and their families are well aware that these must go beyond a focus on ASD symptomatology. Examples include Family Quality of Life (FQOL) (12–15) and ABCX (16–18), both of which consider parental quality of life as an integral component of treatment planning. Regarding the quality of life, researchers emphasize the importance of factors such as happiness, meaning, self-esteem, physical and mental health, social relationships, and goal attainment (19). There are various dimensions within the quality of life, ranging from four to eight or even more (20). In recent years, Shalock’s 8-factor framework (21), which includes domains such as self-determination, social inclusion, interpersonal relationships, rights, material well-being, emotional well-being, physical well-being, and personal development, has gained increasing prominence in studies of QoL among people with intellectual disabilities (20, 22–24), people with visual impairments (25), residents of group homes (26) and other populations.

Of the many quality-of-life assessments available worldwide, only a few are specifically tailored to individuals with ASD or their caregivers. Even fewer have been translated into Polish and psychometrically validated. The currently available translated questionnaires do not focus on individuals with ASD and fail to address the core symptoms of ASD or the challenges experienced by their families. Therefore, there is a significant need for a well-validated Polish-language tool specifically designed to assess the quality of life of families caring for patients with ASD.

### 1.4 Quality of life in autism questionnaire

The Quality of Life in Autism Questionnaire was developed to assess the quality of life experienced by parents of children diagnosed with ASD, aged 2 to 18 (27). It consists of two sections, comprising 48 items, and utilizes a Likert scale for responses. Part A includes 28 items evaluating the parent’s own quality of life and emotional well-being, while Part B contains 20 items assessing the degree of difficulty the parent experiences with specific autism-related behaviours in their child. Although both sections contribute to a comprehensive understanding of the parental experience, they address distinct psychological constructs related to different individuals (parent vs. child) and are therefore typically analysed separately. This questionnaire has shown good psychometric properties and has been validated in several languages, making it a reliable tool for assessing the impact of ASD on parents’ quality of life.

### 1.5 Aims of this study

The first validation was conducted for the English version. This research aims to adapt the original QoLA questionnaire to Polish conditions and to validate the new adaptation using a sample of parents of children diagnosed with ASD. In addition to examining the factorial structure and reliability of the instrument, we seek to assess its concurrent and criterion validity. Based on the existing literature, we formulated several hypotheses. First, we expected a strong negative correlation between parental quality of life (QoLA-A scores) and depressive symptoms, as measured by the Beck Depression Inventory. Second, we hypothesized that quality of life would be lower among single, unemployed parents of children with disabilities who are nonverbal. Finally, we anticipated that lower QoLA-A scores would correlate with reduced material well-being.

## 2 Materials and methods

### 2.1 Translation procedure

The study was approved by the Bioethics Committee of the Medical University of Warsaw (approval number AKBE/232/2023). In accordance with the World Health Organization (WHO) guidelines for the translation and adaptation of psychometric instruments (28), two independent researchers performed the initial forward translation of the questionnaire. Both were native Polish speakers with proficiency in English and familiarity with the relevant terminology in the field. Subsequently, a discussion of the translations was held among the three authors of this manuscript (ŁK, MK, and JŁ), resulting in a single, final version of the questionnaire. In the third step, two translators, both native English speakers fluent in Polish, back-translated the instrument into English. ŁK, MK, and JŁ then reviewed the back translations to identify discrepancies between them and the original tool, leading to the agreement on the final Polish version of the questionnaire.

### 2.2 Participants

Parents of children with ASD, aged 2 to 18 years, participated in the survey. The questionnaire was distributed online through websites and social media platforms that gathered a community of families of children with ASD.

### 2.3 The instrument

The Quality of Life in Autism questionnaire consists of two parts (27). Part A contains 28 questions that assess a parent’s quality of life, with responses recorded on a 5-point Likert scale. Part B lists 20 typical behaviours observed in children with ASD and evaluates how difficult these behaviours are for the parent, using a 5-point Likert scale. The total score of the QoLA questionnaire ranges from 48 to 240 and can also be analysed separately for parts A and B.

To assess the psychometric properties of the QoLA-A scale, we included two additional standardized measures. The Beck Depression Inventory (BDI) was administered to parents to assess depressive symptoms, which are known to impact perceived quality of life. This allowed us to examine the criterion validity of the QoLA-A. In addition, we used the WHOQOL-BREF questionnaire to assess convergent validity, by comparing QoLA-A scores with a general, widely validated measure of quality of life. These instruments were selected to ensure that the scale demonstrates validity both in relation to established psychological constructs and to broader well-being outcomes.

The WHOQOL-BREF, a shortened version of the WHOQOL-100 questionnaire, was developed to assess quality of life (29). It consists of 26 questions covering four domains: physical health, psychological health, social relationships, and environment. Responses are given on a 5-point Likert scale. Calculated and converted scores in each domain range from 4 to 20 points.

The Beck Depression Inventory (BDI) is a self-report questionnaire used to assess the severity of depressive symptoms in adults (30). It consists of 21 closed-ended questions covering hopelessness, irritability, guilt, lack of satisfaction, and somatic symptoms. Responses are scored on a scale from 0 to 3, with higher scores indicating more severe depressive symptoms.

Both measures have been previously translated into Polish and validated. All instruments were completed by the parents; no questionnaires were administered to the children.

### 2.4 Statistical analysis

R, was used to statistically analyze the collected data. Descriptive statistics were used to summarise the demographic characteristics and responses of the participants. In addition, both exploratory factor analyses (EFA) and confirmatory factor analyses (CFA) were conducted using the R psych and lavaan packages to assess the construct validity of the QoLA questionnaire. Cronbach’s alpha coefficients were calculated to assess the internal consistency of QoLA-A and QoLA-B (31). However, as we assumed a multidimensional structure of the inventories, omega h and omega total statistics were also calculated, as they are more appropriate for multidimensional questionnaires (32). To further investigate the nature of the variables influencing the quality of life, structured equation modeling was performed using the R lavaan package. Exploratory factor analysis with Promax rotation was used to examine the structural dimensions of QoLA. Four different methods were used to determine the number of factors: eigenvalues greater than 1 (resulting in 7 factors), Cattell’s scree plot (6 factors), variances accounting for 80% of the variance (13 factors), and the minimum number of factors required for a non-significant chi-square test of model fit (10 factors). In line with Shallock’s model of quality of life, which suggests 8 domains, we aimed for an 8-factor model accounting for 67% of the variance. Items entering the 8 factors are compared against their corresponding Shallock’s domains in **Table 1**.

**Table 1:** Comparison of our CFA 8-factor structure of the QoLA-A and Schalock’s 8 domains of quality of life.

Factor	Items	Domain	Schalock’s domain
1	6, 7, 8, 9	Social relationships	Interpersonal relationships
2	5, 13, 18, 19, 20, 21	Self-directedness	Self-determination
3	2, 4, 22	Psychological health	Emotional well-being
4	10, 11, 12	Financial security	Material well-being
5	14, 15, 17	Physical health	Physical well-being
6	23, 24, 28	Community	Social inclusion
7	1, 3	General happiness	
8	16, 25, 26, 27	Security	Rights

## 3 Results

### 3.1 Sociodemographic characteristics of the sample

The study sample consisted of 372 respondents, predominantly mothers, with an average age of 44.9 years. **Table 2** below summarizes key demographic and socioeconomic characteristics, including place of residence, education level, employment status, financial situation, and marital status.

**Table 2:** Summary of sociodemographic characteristics of the sample.

Characteristic	N (%)
**Age (mean)**	44.9 years
**Total respondents**	372 (100%)
Gender	
Female	350 (95.89%)
Male	15 (4.11%)
Place of residence	
Towns over 100,000 inhabitants	167 (45.75%)
Towns below 100,000 inhabitants	122 (33.43%)
Rural areas	76 (20.82%)
Education	
Higher	271 (73.84%)
Secondary	81 (22.07%)
Vocational	12 (3.27%)
Primary	3 (0.82%)
Employment status	
Full-time	181 (49.32%)
Part-time	50 (13.62%)
Reciving caregiver allowance	36 (9.81%)
Unemployed	100 (27.25%)
Financial status	
Very good	45 (12.26%)
Good	118 (31.15%)
Medium	167 (45.50%)
Poor	32 (8.72%)
Very poor	5 (1.37%)
Marital status	
Married	262 (71.78%)
Non-marital relationship	37 (10.14%)
Single	18 (4.93%)
Divorced	45 (12.33%)
Widowed	3 (0.82%)

Descriptive analyses were conducted to characterize the study population in terms of demographic, clinical, and psychological variables. Most children in the sample were boys, and the average age was approximately 10 years. Nearly half of the respondents had two children. A large majority of the children communicated using words, while approximately one in five were reported to have an intellectual disability. More than half of the responding parents had received an autism spectrum disorder (ASD) diagnosis themselves.

**Figure 1:**
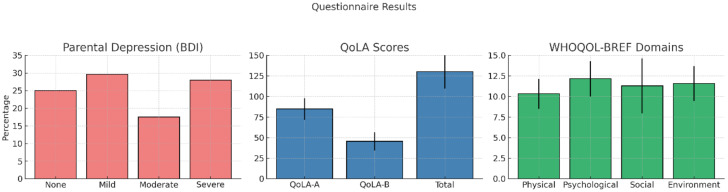
Mean scores on the QoLA-A and QoLA-B scales, WHOQOL-BREF domains, and levels of depressive symptoms (BDI) among participating parents. Error bars represent standard deviations.

Mean quality of life scores as measured by the QoLA-A and QoLA-B scales are presented in **Figure 1**, along with WHOQOL-BREF domain scores. The distribution of parental depressive symptoms is also summarized.

### 3.2 QoLA part A

#### 3.2.1 Validity

A number of analyses were carried out to provide comprehensive evidence of the QoLA questionnaire’s construct validity.

**Figure 2:**
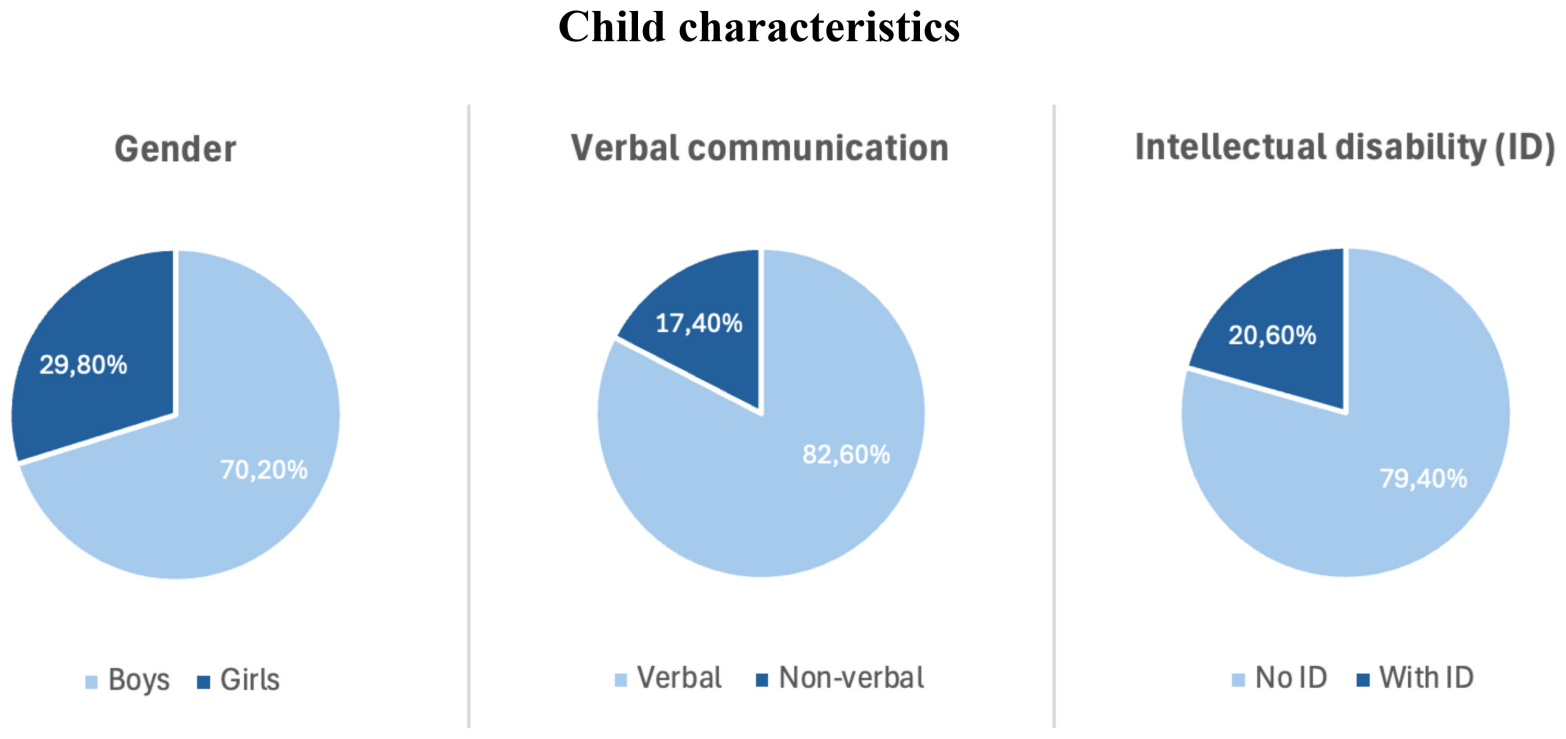
Distribution of child characteristics in the sample, including gender, verbal communication, and presence of intellectual disability.

To confirm the 8-factor model, a Confirmatory Factor Analysis was conducted. The model displayed satisfactory fit measures: Root Mean Square Error of Approximation = 0.056 and Comparative Fit Index = 0.92. The resultant model, illustrated in **Figure 2**, covers all the factors proposed by Shalock except for “Personal growth,” which was replaced with “General well-being.” In general, the factor analysis suggested that the Polish version of QoLA part A is a tool that evaluates at least seven out of eight domains of quality of life. The correlations between the latent variables were moderate to high, indicating a common underlying factor and allowing for total score calculations (see Reliability section). The CFA findings are presented in **Table 3**.

**Table 3:** Factor loadings of the 8-dimensional CFA model of QoLA-A, CR, Composite Reliability; AVE, Average Variance Extracted.

Construct	Item	Factor loading	Cronbach’s α	CR/AVE
Social relationships	QoLA_A_6	0.755	0.815	0.816/0.533
	QoLA_A_7	0.72	0.815	
	QoLA_A_8	0.529		
	QoLA_A_9	0.873		
Self-directedness	QoLA_A_5	0.75	0.819	0.821/0.436
	QoLA_A_13	0.665	0.819	
	QoLA_A_18	0.652		
	QoLA_A_19	0.749		
	QoLA_A_20	0.597		
	QoLA_A_21	0.521		
Psychological health	QoLA_A_2	0.634	0.68	0.7/0.439
	QoLA_A_4	0.748	0.68	
	QoLA_A_22	0.597		
Financial security	QoLA_A_10	0.873	0.776	0.798/0.588
	QoLA_A_11	0.442	0.776	
	QoLA_A_12	0.898		
Physical health	QoLA_A_14	0.896	0.725	0.769/0.535
	QoLA_A_15	0.582	0.725	
	QoLA_A_17	0.68		
Community	QoLA_A_23	0.659	0.645	0.655/0.411
	QoLA_A_24	0.823	0.645	
	QoLA_A_28	0.348		
General happiness	QoLA_A_1	0.851	0.877	0.85/0.739
	QoLA_A_3	0.868	0.877	
Security	QoLA_A_16	0.551	0.715	0.725/0.402
	QoLA_A_25	0.548	0.715	
	QoLA_A_26	0.746		
	QoLA_A_27	0.668		

R-Pearson correlation values of QoLA part A and four domains of WHOQoL-BREF are shown in **Table 4**.

**Table 4:** Correlation coefficients between the 4 domains of WHOQoL-BREF and QoLA-A.

WHOQoL-BREF	r-Pearson correlation	Interpretation
Domain 1 Somatic	0.58	Moderate
Domain 2 Psychological	0.66	Moderate
Domain 3 Social	0.66	Moderate
Domain 4 Environment	0.75	High

Correlation strength was interpreted as follows: coefficients from 0.30 to 0.7 were considered moderate, and those ≥ 0.70 were considered strong.

The moderate to high correlations between QoLA-A scores and the four domains of the WHOQoL-BREF suggest good criterion validity. A moderate positive correlation was found between QoLA-A scores and domains related to social relationships, somatic health, and psychological well-being. In addition, a high positive correlation was observed between QoLA-A scores and the environmental domain. These correlations suggest that the QoLA-A effectively measures constructs similar to those of the WHOQoL-BREF in assessing quality of life, validating its use as a reliable tool for assessing individuals’ well-being across domains.

To test concurrent validity, the correlation between QoLA-A scores and the Beck Depression Inventory (BDI) was examined. As expected, the correlation coefficient was –0.71, indicating a strong negative relationship between parental quality of life and depressive symptoms. This finding supports the scale’s criterion validity by demonstrating its sensitivity to psychological distress, a well-established factor associated with reduced wellbeing in parents of children with ASD.

In addition, regression analysis was conducted to assess the relationship between QoLA-A scores and sociodemographic characteristics. The results showed that single parenthood reduced QoLA-A scores by 4.8 points, unemployment by 7.22 points, and having a child with an intellectual disability by 3.9 points. Notably, having a child who could not communicate verbally did not have a statistically significant effect on the QoLA-A score. These results are consistent with theoretical expectations and support the scale’s validity in reflecting the impact of known risk factors on parental quality of life.

#### 3.2.2 Reliability

The reliability of the QoLA part A was found to be high, as indicated by a Cronbach’s alpha coefficient of 0.93. There was no effect on the alpha value when any item was excluded. To take account of the multidimensional nature of the QoLA part A, we also assessed its reliability using the hierarchical omega coefficient (omega h), which was 0.77 - slightly lower than the alpha coefficient but still within an acceptable range (above 0.7). The common factor explained 47% of the total variance. The total omega was 0.95. These results confirm that the QoLA Part A has satisfactory reliability and can effectively measure the quality of life of parents of autistic children, both as a single construct and as an 8-dimensional model. Based on these findings, using the total score as a measure of quality of life is reasonable.

### 3.3 QoLA part B

#### 3.3.1 Validity

The second part’s exploratory factor analysis resulted in a five-dimensional model with an RMSEA of 0.044, which is below the threshold of 0.05 (**Table 5**). Models with fewer dimensions are also needed to fit the data adequately. The five-dimensional model accounted for only 42.7% of the variance, so even these dimensions were insufficient to explain most of the variance in the scores. Based on the data and findings, there was no compelling need to conduct a confirmatory factor analysis.

**Table 5:** Factor loadings of the 5-dimensional model of QoLA-B from EFA.

QoLA item/Factor	*Factor1*	*Factor2*	*Factor3*	*Factor4*	*Factor5*
QoLA.B.1	-0.30940	0.10284	0.71484	0.07586	0.06469
QoLA.B.2	-0.06572	-0.09097	0.87548	-0.00678	0.00870
QoLA.B. 3	0.36410	-0.18937	0.23775	0.12194	0.26459
QoLA.B.4	-0.14596	-0.09698	0.32058	0.38506	0.05491
QoLA.B.5	0.02079	-0.03967	0.00314	0.64635	0.04662
QoLA.B.6	-0.00889	0.31603	-0.00219	-0.06738	0.60069
QoLA.B.7	0.10836	0.10274	0.05090	-0.09827	0.49504
QoLA.B.8	0.03106	0.56853	0.14536	-0.12861	0.08957
QoLA.B.9	0.20054	0.33946	0.21556	-0.13451	-0.04467
QoLA.B.10	-0.12399	0.74392	-0.18543	0.10724	0.20826
QoLA.B.11	-0.11551	0.54811	-0.15437	0.26503	0.17806
QoLA.B.12	0.33482	-0.07529	0.16327	0.13243	0.27880
QoLA.B.13	0.47412	0.17801	-0.17630	0.22218	-0.01977
QoLA.B.14	0.16489	0.50881	0.15718	-0.08246	-0.05629
QoLA.B.15	0.82943	-0.00151	-0.18172	-0.03190	-0.08303
QoLA.B.16	0.73347	0.01963	-0.10198	-0.06253	0.13849
QoLA.B.17	0.35460	0.11262	0.26796	0.09188	-0.18337
QoLA.B.18	0.64639	-0.11345	-0.00415	0.04282	0.14954
QoLA.B.19	0.11356	0.07178	0.05736	0.56086	-0.19296
QoLA.B.20	-0.06091	0.12418	-0.03977	0.62018	-0.06341

The analysis suggests that the validity of the second part of the QoLA is unsatisfactory, as it covers different aspects of autism spectrum disorder but accounts for only a limited amount of score variability.

#### 3.3.2 Reliability

The reliability of the QoLA part B was assessed using Cronbach’s alpha coefficient, which was 0.86. The removal of one item did not affect the reliability. The calculated omega h was 0.55, below the accepted threshold of 0.7. On the other hand, the total omega (i.e., the five factors plus the general factor) was 0.9, which is very good.

Following the guidelines of authors who suggest using omega total rather than Cronbach’s alpha for multidimensional scales (33), we conclude that the QoLA Part B is a reliable measure of a 5-dimensional construct but a weak measure of a single construct.

### 3.4 QoLA – B: externalizing behaviours

**Figure 3:**
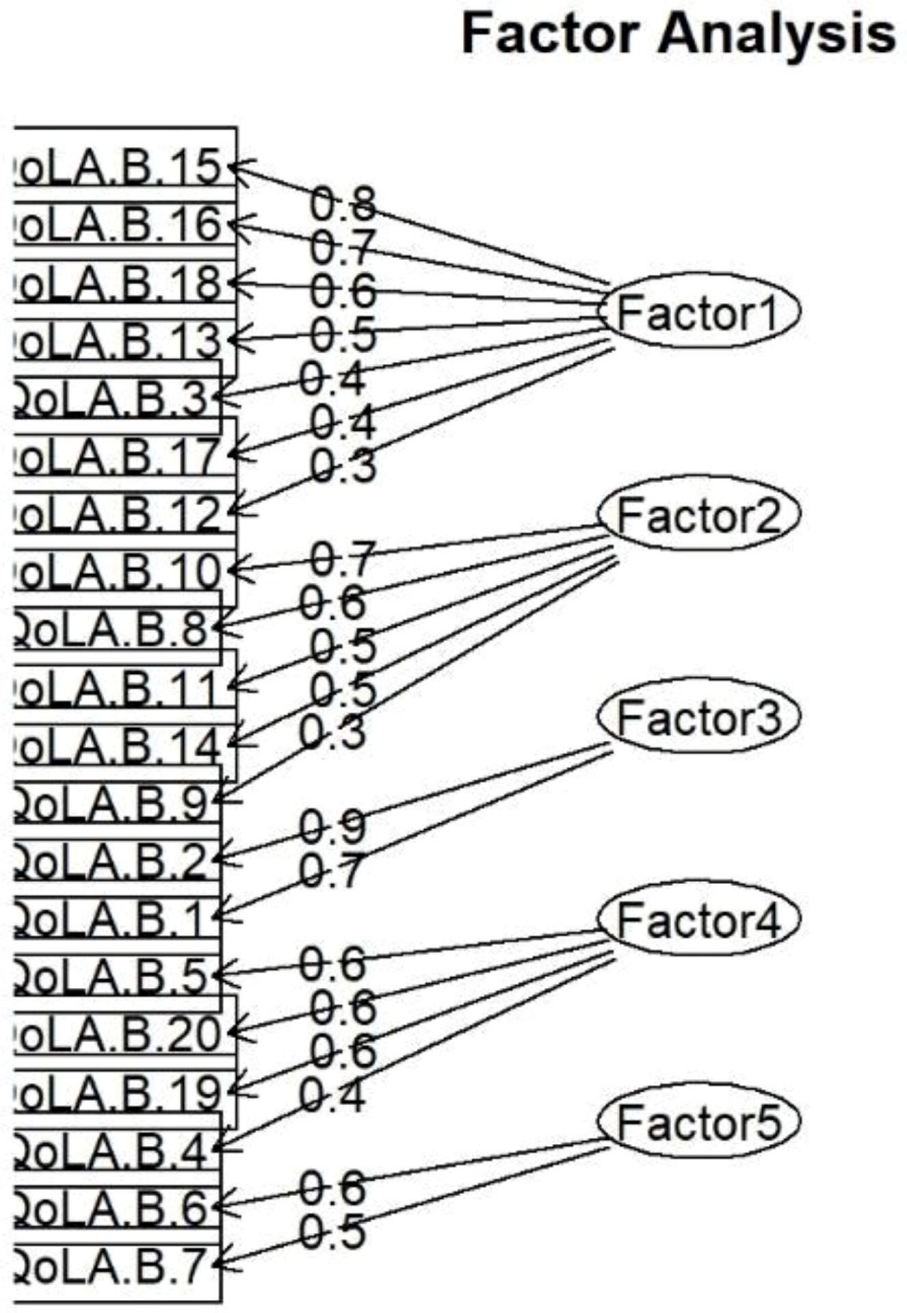
The 5-factor model with factor loadings from EFA, QoLA-B.

Although QoLA-B did not prove to be an adequate measure of the impact of ASD symptoms on quality of life in general, we decided to extract items from Factor 1 (see **Figure 3**), which covers externalizing behaviors, for further analysis. Cronbach’s alpha coefficient estimated its reliability at 0.79, justifying its use to measure externalizing behaviors.

### 3.5 Comparisons and regression models

**Figure 4:**
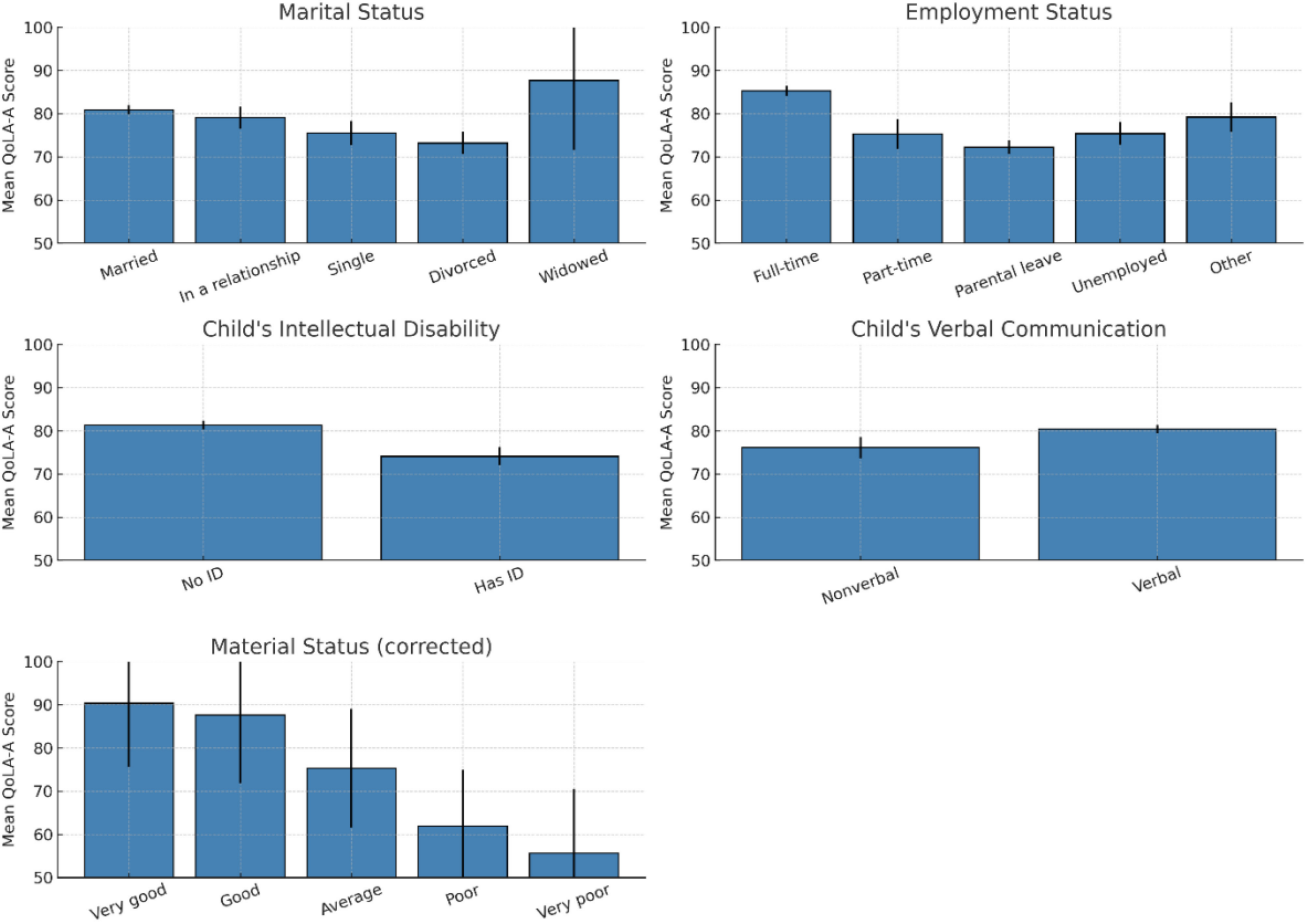
Comparisons of mean QoLA-A score by groups. Error bars represent standard errors of the mean.

Comparison of means revealed that parents who were married, employed full-time, and reported a higher material status tended to report higher levels of quality of life. Similarly, parents of verbally communicative children and those without an intellectual disability in the child also reported higher QoLA-A scores (see **Table 6**). These findings are consistent with intuition and prior research and provide further support for the construct validity of the instrument. Notably, widowed participants (N = 3) reported relatively high QoLA-A scores; however, this subgroup was extremely small, and the high standard deviation suggests substantial variability. Therefore, this result should be interpreted with caution, as it may reflect individual characteristics or random variation rather than a systematic pattern. The comparisons are presented in **Figure 4**.

**Table 6:** Results of univariate regression analyses of independent variables on QoLA-A scores.

Independent variables	Beta coefficient	p-value
Single parent	-4.7911	< 0.006
Unemployed	-7.2278	< 0.001
Child disabled	-3.9036	< 0.026
The child uses communicative speech	0.1199	> 0.951

A number of regression models were used to further elucidate the factors influencing the quality of life of the participants in our study. A backward stepwise multiple regression analysis, which systematically removes variables that do not contribute significantly to the model, was applied to the initial set of nine explanatory variables. Initially including nine explanatory variables - the presence of communicative speech in the child, the presence of intellectual disability, Beck Depression Scale scores, parental age, child age, number of children living in the household, employment status, marital status, and externalizing behaviors exhibited by the child - the analysis eventually reduced the model to include only four variables: Beck Depression Scale scores, employment status, material status, and externalizing behaviors exhibited by the child. The relevant coefficients of this model are shown in **Table 7**. The final model was statistically significant, F = 189.1, df(4, 356), p < 2.2 x 10-16, and explained 68% of the variance in the scores (R2 = 0.68, adj. R2 = 0.68).

**Table 7:** The end model of the stepwise backward multiple regression.

Variable	Beta coefficient	Standard error	p-value
BDI score	-0.99	0.05	2.2 x 10^-16^
Employment	-1.23	0.39	0.00173
Material status	-4.38	0.63	1.96 x 10^-11^
Child’s externalizing problems	0.40	0.11	0.0004

The independent variable is the QoLA-A score.

We then used this model as the basis for structured equation modeling, where a latent variable, ‘quality of life,’ was defined as a composite of QoLA-A and WHOQOL dimensions 1,2,3 and 4 scores. Model 0 included the four explanatory variables obtained by stepwise regression. Model 1 included verbal communication and intellectual disability as mediators of parental depression. Model 2 included the number of children in the household, parental education, intellectual disability, and employment as mediators of material status. Material status was positioned as a higher-order variable based on both theoretical and empirical considerations. Conceptually, financial well-being is an upstream determinant of key life circumstances, such as educational attainment, employment status, and family size. These factors are, in turn, known to influence parental quality of life. By modeling material status as an exogenous variable with potential mediating pathways, we aimed to better understand the mechanisms through which financial conditions shape well-being. This structure was further supported by the results of the structural equation modeling, which identified employment and education as significant mediators of the material status–quality of life relationship. In model 1, no significant mediators were found; in model 2, the mediating effects of employment and education on the effect of material status on quality of life were statistically significant (see **Table 8**). The other mediators were not significant.

**Table 8:** Mediation effects and the total effect of material status on quality of life.

Mediators	Estimate	Std. Err	z-value	P(>|z|)	Std. lv	Std. all
Intellectual disability	-0.001	0.082	-0.012	0.991	-0.000	-0.000
Number of children	-0.059	0.055	-1.065	0.287	-0.004	-0.003
Employment	-0.247	0.111	-2.234	0.025	-0.016	-0.014
Education	0.320	0.131	2.441	0.015	0.021	0.018
Total	-4.659	0.531	-8.778	0.000	-0.304	-0.262

Model 2 provided the greatest increase in R2 and the greatest decrease in RMSEA, bringing it below 0.1, which is considered acceptable for a model, while keeping CFI slightly above 0.9, which is still considered acceptable (see **Table 9**). Therefore, we chose model 2 as the best representation of the data obtained. Moreover, Model 2 demonstrated the best overall fit among the three tested models. This suggests that placing material status in a higher-level position provides a more accurate representation of the data. **Table 8** lists the 4 mediation effects of the model, 2 of which were statistically significant.

**Figure 5:**
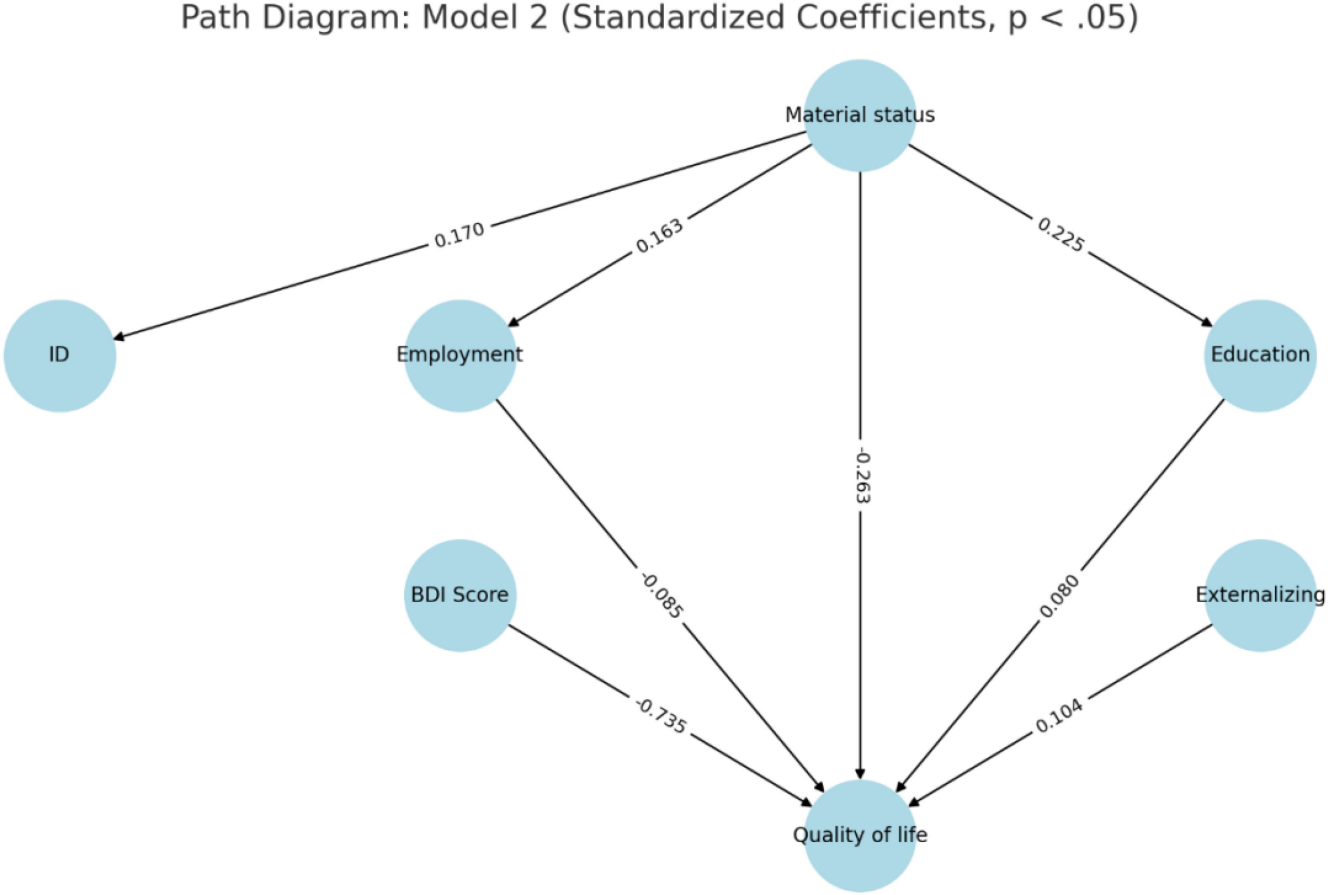
Path diagram of Model 2 with standardized coefficients of statistically significant effects (p <.05). Model 2 tested the mediating role of employment, education, intellectual disability (ID), and number of children in the household in the relationship between material status and parental quality of life (QoL). Only paths with p-values below.05 are shown. Material status was modeled as an exogenous variable based on both theoretical rationale and empirical model fit improvements ΔR², .008; ΔRMSEA, –.023). BDI, Beck Depression Inventory.

**Table 9:** Comparison of 3 models.

Model/Fit change indices	R^2^	ΔR^2^	Δχ^2^	ΔCFI	ΔRMSEA
Model 0	0.834	–	132.142	0.929	0.120
Model 1	0.835	+0.001	+90.138	-0.042	-0.001
Model 2	0.842	+0.008	+78.07	-0.027	-0.023

Model 0: no mediators; Model 1: verbal communication and intellectual disability as mediators of depression; Model 2: Number of children, employment, education, and intellectual disability as mediators of material status. The goodness of fit measures and their changes relative to model 0 are provided.

In model 2, QoL is directly predicted by BDI scores, child externalizing problems, parental education, employment, and material status. Indirectly, poorer material status predicts lower education and employment, which worsens quality of life. The poorer material status also predicts intellectual disability, but this variable did not affect the quality of life. The mediation paths are presented in **Figure 5** and coefficients are listed in **Table 10**.

**Table 10:** Path analysis of model 2 with standardized coefficients of statistically significant effects (p <.05).

Model factors/Model parameters	Estimate	Std. Err	z-value	P(>|z|)	Std. lv	Std. all
Quality of life						
ID	-0.012	1.018	-0.012	0.991	-0.001	-0.000
Child. in house.	0.839	0.471	1.781	0.075	0.055	0.048
Employment	-0.997	0.316	-3.155	0.002	-0.065	-0.085
Education	2.146	0.732	2.931	0.003	0.140	0.080
BDI Score	-1.033	0.047	-21.992	0.000	-0.067	-0.735
Material status	-4.672	0.542	-8.614	0.000	-0.305	-0.263
Externalizing	0.331	0.091	3.628	0.000	0.022	0.104
ID						
Material status	0.080	0.024	3.305	0.001	0.080	0.170
Child. in house.						
Material status	-0.070	0.053	-1.329	0.184	-0.070	-0.069
Employment						
Material status	0.248	0.078	3.163	0.002	0.248	0.163
Education						
Material status	0.149	0.034	4.409	0.000	0.149	0.225

Results of model 2. ID = Intellectual disability, Child. In house. = number of children in the household.

## 4 Discussion

### 4.1 Summary of findings

In this study, we conducted several analyses to evaluate the measurement properties of the QoLA questionnaire using a sample of parents of children with ASD. In addition, we aimed to identify factors that influence their quality of life.

Part A of the questionnaire showed satisfactory reliability as measured by Cronbach’s alpha, omega h, and omega total. To investigate the structure of QoLA-A, we used exploratory factor analysis and explored different methods to determine the number of factors. Ultimately, an 8-factor model aligned with Schalock’s quality of life domains was confirmed by confirmatory factor analysis, which showed satisfactory goodness of fit. Criterion validity was established by assessing correlations between QoLA Part A scores and the WHOQOL-BREF and the Beck Depression Inventory. The results showed moderate to high correlations, supporting its relevance for different aspects of well-being. Based on the results, it is concluded that the QoLA-Part A is a valid and reliable measure of quality of life in people with autistic spectrum disorders. The scores also correlated moderately to strongly with established predictors of QoL, such as depression, marital status, material status, and unemployment. This is consistent with the questionnaire adaptations from Turkey (34), Iran (35), Bangladesh (36), and Malaysia (37).

Part B’s reliability was confirmed, but exploratory factor analysis revealed a 5-dimensional structure that explained less than 50% of the variance in scores, failing to prove that the questionnaire measures ASD symptomatology as commonly described. This contrasts findings from Turkey (34) and transcultural studies (38), where a three-dimensional structure explaining over 60% of score variance is consistently reported. This suggests that cultural differences may play a role in parents’ perceptions of ASD symptoms in their children. Indeed, this has been confirmed in a cross-cultural comparison showing that culture plays an important role in reporting symptoms related to repetitive behaviors, with Polish and US parents more likely to endorse these items than parents from other countries (39). This may have led to items related to these behaviors being split into multiple dimensions. Nevertheless, caution should be exercised when using the QoLA Part B to assess parent-reported ASD symptomatology in Poland.

Although the overall Cronbach’s alpha and omega coefficients justify the use of the QoLA-B as a reliable measure of the five dimensions, the low omega h value, the failure to capture the predicted dimensionality, and the low explanatory power of the questionnaire’s five-dimensional model raise serious doubts about the use of the inventory to measure the impact of ASD symptoms on quality of life in Poland.

### 4.2 Child-related factors of parental quality of life

Our results showed that of the child-related variables included in the study - intellectual disability, lack of verbal communication, child’s age, gender, and externalizing problems - only the latter was a statistically significant predictor of parents’ quality of life. This seemed puzzling, as intellectual disability and lack of verbal communication could potentially be a major burden for carers. However, other researchers have also found that in families of children with ASD, externalizing problems such as oppositional defiant disorder and aggression are more strongly associated with poorer quality of life scores than communication difficulties (40). Similarly, ASD subtype or severity was not found to influence maternal distress (41) or quality of life (42) when child behavioral problems were taken into account. Other data also suggest a limited or no effect of child-related variables on parental QoL (43). Intellectual disability had a limited or no effect on parental QoL in some studies (41, 44, 45). Physical disability did not worsen maternal QoL (46). Communication problems were found to hurt QoL, but parental optimism may act as a mediator (47). A meta-regression study found neither IQ nor ASD severity predicted adult QoL, but social functioning did (48). On the other hand, some studies report a negative impact of autism severity on family quality of life (17, 49–51) (but only for mothers, fathers reported an inverse relationship), even compared to other disabilities, including neurological ones (52). The reason for these conflicting results may be that intellectual disability (ID) is a spectrum ranging from mild to severe, with 85% of those diagnosed being mildly impaired (1, 53). We did not ask respondents to indicate the level of intellectual disability of their children, but we can assume that most were mildly impaired. In one paper, we found that parents of mildly mentally disabled children reported a quality of life similar to that of neurotypical children, in contrast to parents of moderately intellectually disabled children (12). Another plausible explanation for this phenomenon is that psychiatric diagnoses may be less able to accurately predict parents’ quality of life than functional diagnoses and the child’s level of autonomy. This assertion implies that factors related to the individual’s functional abilities and the degree of independence they can exercise may have greater predictive power in determining overall quality of life, as suggested by existing research (51, 52). Finally, our respondents were almost exclusively mothers, which means that child-related variables may have a greater impact on fathers’ quality of life.

### 4.3 Social factors of parental quality of life

Our model, including the social variables, explained over 80% of the variance, which is very high. It should be noted, however, that this high percentage may be influenced by the fact that some QoLA-A questions deal with material status, which contributes to an inflated covariance. This implies that the social variables included in our model play a crucial role in predicting parents’ quality of life. We found that lower material status, employment status, and educational attainment predicted lower quality of life.

In our univariate analyses, single parenthood was initially found to predict lower quality of life. However, when additional variables were included in the analysis, this effect was attenuated, suggesting a mediating influence of these additional factors. This observation aligns with findings from previous studies in which single parenthood was similarly dependent on a range of socioeconomic factors (52). This suggests that while single mothers may have a lower reported quality of life, this outcome is predominantly due to a lack of material resources and support from a partner rather than their marital status per se.

The presence of additional offspring had no significant effect on the outcome, which differs from previous studies suggesting that an increased number of children correlates with improved quality of life (53). This finding is consistent with existing research (18, 54). The potential influence of neurodevelopmental diagnoses in siblings warrants consideration in this context.

Although some studies do not report on the influence of material status on quality of life (54, 55), most do (13, 15, 56–58). A Greek study comparing the influence of the COVID-19 pandemic and the financial crisis of 2014–5 provides interesting insights, showing that the economic crisis worsened parental quality of life more than the pandemic, highlighting the role of financial stability (59). This is in line with the findings of other studies.

### 4.4 Maternal depression and mother-related variables

We measured levels of depression in our sample and found 28% of the respondents were experiencing severe depression and only 25% had no symptoms. Additionally, maternal depression deteriorated their quality of life even when socio-economic variables were taken into an account. This is in line with other evidence showing that the prevalence of depression in mothers of autistic children is high and affects their quality of life as well as ASD symptom severity in children (52, 60). A study conducted among 409 parents of children with ASD in China, revealed that 18.8% exhibited significant depressive symptoms, with notably higher occurrence observed among families experiencing high economic burden, caring for a child with limited verbal communication, expressing dissatisfaction with their current marital status and facing unemployment (58).

We did not find any moderators of the influence of maternal depression on quality of life, however other studies point to personality traits like neuroticism (61). Spousal support may serve as a significant factor (62, 63) although findings exhibit variability (62). It is plausible that beyond the mere presence of a spouse, the quality of the interpersonal relationship within the dyad plays a critical role (13). Perception of the child by immediate neighbours has also been shown to affect maternal depression (64). Extensive investigation has been dedicated to examining maternal coping mechanisms. Research indicates that a task-oriented approach and ego-resiliency, loosely defined as the ability to withstand stress, are robust predictors of enhanced maternal quality of life, whereas an emotion-focused coping style demonstrates a contrary effect (63).

### 4.5 Strengths and limitations of our study

A key strength of our survey is the relatively large sample size, which increases our findings’ statistical power and generalisability. However, as the survey was distributed online via social media platforms, it reached a limited demographic group. This distribution method may have yet to capture a broad and diverse cross-section of the population, potentially bias our results and limiting the scope of our conclusions. Future studies should include a higher representation of fathers and participants with a more diverse educational status. In addition, eligibility in our questionnaire was based only on parental reports of ASD. It is possible that not all families had children diagnosed with ASD using scientifically accepted methods.

## 5 Conclusions

In conclusion, the validation of the questionnaire showed that the QoLA Part A is suitable for use in Polish settings and well reflects the quality of life of parents of children with ASD. However, Part B should be used cautiously when measuring ASD-related quality of life.

Based on the results of the multivariate analysis, we suggest that policymakers focus on improving the material status of families raising children with ASD as a key pathway to enhancing parental quality of life. In addition to direct financial assistance, such as targeted benefits or tax relief, this could include active measures such as subsidized employment opportunities, flexible work arrangements, and access to vocational training—particularly for mothers, who often bear the greatest caregiving burden. In addition, early screening for signs of depression in parents and appropriate psychological and medical support should be provided. In addition, behavioral therapy and parent counseling for externalizing and internalizing problems should be offered. Increasing the child’s autonomy and facilitating their participation in a normal school environment is also recommended, as parents with a child who attended school and those with a child who had leisure activities in a normal environment reported less stress and a better quality of life (60).

On the other hand, we found no compelling evidence, either in this study or in the literature, to recommend increasing the amount of ASD-specific therapeutic activities as a means of improving maternal quality of life. An over-concentration of individual work with the child to alleviate ASD symptoms may decrease parents’ quality of life (64). Some research has found that attending autism support groups and parent training had no significant effect on quality of life (57) or that parents who attended reported lower quality of life than those who did not (45).

Another reason to focus on parents’ quality of life is that data suggests that while behavior does not influence maternal well-being, the reverse is true (54). Parents who attend autism support groups report lower quality of life.

## Data Availability

The raw data supporting the conclusions of this article will be made available by the authors, without undue reservation.
